# COVID-19 in non-hospitalised adults caused by either SARS-CoV-2 sub-variants Omicron BA.1, BA.2, BA.4/5 or Delta associates with similar illness duration, symptom severity and viral kinetics, irrespective of vaccination history

**DOI:** 10.1371/journal.pone.0294897

**Published:** 2024-03-21

**Authors:** Hermaleigh Townsley, Joshua Gahir, Timothy W. Russell, David Greenwood, Edward J. Carr, Matala Dyke, Lorin Adams, Murad Miah, Bobbi Clayton, Callie Smith, Mauro Miranda, Harriet V. Mears, Chris Bailey, James R. M. Black, Ashley S. Fowler, Margaret Crawford, Katalin Wilkinson, Matthew Hutchinson, Ruth Harvey, Nicola O’Reilly, Gavin Kelly, Robert Goldstone, Rupert Beale, Padmasayee Papineni, Tumena Corrah, Richard Gilson, Simon Caidan, Jerome Nicod, Steve Gamblin, George Kassiotis, Vincenzo Libri, Bryan Williams, Sonia Gandhi, Adam J. Kucharski, Charles Swanton, David L. V. Bauer, Emma C. Wall

**Affiliations:** 1 The Francis Crick Institute, London, United Kingdom; 2 National Institute for Health Research (NIHR) University College London Hospitals (UCLH) Biomedical Research Centre and NIHR UCLH Clinical Research Facility, London, United Kingdom; 3 Centre for Mathematical Modelling of Infectious Diseases, London School of Hygiene & Tropical Medicine, London, United Kingdom; 4 Worldwide Influenza Centre, The Francis Crick Institute, London, United Kingdom; 5 University College London, London, United Kingdom; 6 Genotype-to-Phenotype UK National Virology Consortium (G2P-UK); 7 London Northwest University Healthcare NHS Trust, London, United Kingdom; 8 Camden and North West London NHS Community Trust, London, United Kingdom; 9 Department of Infectious Disease, St Mary’s Hospital, Imperial College London, London, United Kingdom; Lagos State University, NIGERIA

## Abstract

**Background:**

SARS-CoV-2 variant Omicron rapidly evolved over 2022, causing three waves of infection due to sub-variants BA.1, BA.2 and BA.4/5. We sought to characterise symptoms and viral loads over the course of COVID-19 infection with these sub-variants in otherwise-healthy, vaccinated, non-hospitalised adults, and compared data to infections with the preceding Delta variant of concern (VOC).

**Methods:**

In a prospective, observational cohort study, healthy vaccinated UK adults who reported a positive polymerase chain reaction (PCR) or lateral flow test, self-swabbed on alternate weekdays until day 10. We compared participant-reported symptoms and viral load trajectories between infections caused by VOCs Delta and Omicron (sub-variants BA.1, BA.2 or BA.4/5), and tested for relationships between vaccine dose, symptoms and PCR cycle threshold (Ct) as a proxy for viral load using Chi-squared (χ2) and Wilcoxon tests.

**Results:**

563 infection episodes were reported among 491 participants. Across infection episodes, there was little variation in symptom burden (4 [IQR 3–5] symptoms) and duration (8 [IQR 6–11] days). Whilst symptom profiles differed among infections caused by Delta compared to Omicron sub-variants, symptom profiles were similar between Omicron sub-variants. Anosmia was reported more frequently in Delta infections after 2 doses compared with Omicron sub-variant infections after 3 doses, for example: 42% (25/60) of participants with Delta infection compared to 9% (6/67) with Omicron BA.4/5 (χ^2^ P < 0.001; OR 7.3 [95% CI 2.7–19.4]). Fever was less common with Delta (20/60 participants; 33%) than Omicron BA.4/5 (39/67; 58%; χ^2^ P = 0.008; OR 0.4 [CI 0.2–0.7]). Amongst infections with an Omicron sub-variants, symptoms of coryza, fatigue, cough and myalgia predominated. Viral load trajectories and peaks did not differ between Delta, and Omicron, irrespective of symptom severity (including asymptomatic participants), VOC or vaccination status. PCR Ct values were negatively associated with time since vaccination in participants infected with BA.1 (β = -0.05 (CI -0.10–0.01); P = 0.031); however, this trend was not observed in BA.2 or BA.4/5 infections.

**Conclusion:**

Our study emphasises both the changing symptom profile of COVID-19 infections in the Omicron era, and ongoing transmission risk of Omicron sub-variants in vaccinated adults.

**Trial registration:**

NCT04750356.

## Introduction

COVID-19 causes a wide range of symptoms in humans; recognition of this diversity now forms the core of global public health messaging, including in the United States, where the Centers for Disease Control and Prevention (CDC) recognise a set of eleven possible symptoms (CDC, 2022) [1]. The emergence of new SARS-CoV-2 variants of concern (VOCs) with substantially different properties such as innate immune antagonism [2] and tissue tropism [3, 4]^,^ has occurred despite widespread vaccination that induces durable immune responses to these variants [5–7]. While vaccination has led to dramatic reductions in hospitalisation and deaths from COVID-19, infection and transmission are less affected by vaccination [8–12]. High numbers of COVID-19 cases caused by the Omicron BA.1 and BA.2 sub-variants in vaccinated individuals were reported across national surveillance systems between December 2021-May 2022 [13], despite a major booster vaccination campaign.

Early reports of Omicron BA.1 infection from South Africa in December 2021 suggested this VOC caused a less-severe clinical disease, as measured by crude outcomes of hospitalisation and mortality rates; in the context of highly vaccinated European populations, similar trends have been reported [8, 9, 14]. While these data are encouraging, they do not account for the significant ongoing-impact of community COVID-19 infections in non-hospitalised adults, with attendant risks of onward transmission and burden on healthcare, particularly for clinically extremely vulnerable individuals (CEV) [15] and those developing post-COVID syndrome (PCS) [16–18]. One study of household transmission in the UK found that while viral kinetics were altered by vaccination, secondary attack rates were similar across VOCs [11]. Furthermore, few studies have prospectively examined relationships between symptoms and VOC infection in non-hospitalised adults, reporting changes in symptoms of COVID-19 between VOCs, but have neither captured asymptomatic infections or nor controlled for time since last vaccine dose, and thus waning immunity [11, 19, 20].

The UK’s NHS COVID-19 guidance was changed in April 2022, at a time when the UK was transitioning from Delta and BA.1 infections, into BA.2 and then BA.4/BA.5 infections, and third vaccine doses had been widely administered (https://www.nhs.uk/conditions/covid-19/covid-19-symptoms-and-what-to-do/). The list of cardinal symptoms was expanded and, self-isolation based on the presence of fever or symptom severity advised. While self-isolation remains in the guidelines, the recommended isolation periods are significantly shorter (3–5 days, with advice to avoid large crowds or contact with clinically-vulnerable individuals for up to 10 days) [1, 21]. The guidance also removed the explicit/general recommendation for the use of non-pharmaceutical interventions (NPIs) and testing following COVID symptoms in parallel with withdrawal of free tests. These guidelines remain in active use in the UK. We investigated if this symptom-based guidance, developed from reports of Alpha and Delta infections in unvaccinated individuals was appropriate for Omicron BA.1, BA.2 and BA.5. We compared symptom profiles and viral load trajectories between healthy, vaccinated adults infected with SARS-CoV-2 variants Delta, BA.1, BA.2 and BA.4/5, stratifying the cohorts by vaccine doses and time since last dose.

## Materials and methods

### Description of the clinical cohort

We analysed data from participants in the University College London Hospitals (UCLH)-Francis Crick Institute *Legacy* study cohort (NCT04750356), who reported a positive SARS-CoV-2 test either through asymptomatic occupational screening or symptom-based testing. The *Legacy* study was established in January 2021 to track serological responses to vaccination during the national COVID-19 vaccination programme in a prospective cohort of healthy staff volunteers.

### Inclusion and exclusion criteria

**Fig 1:**
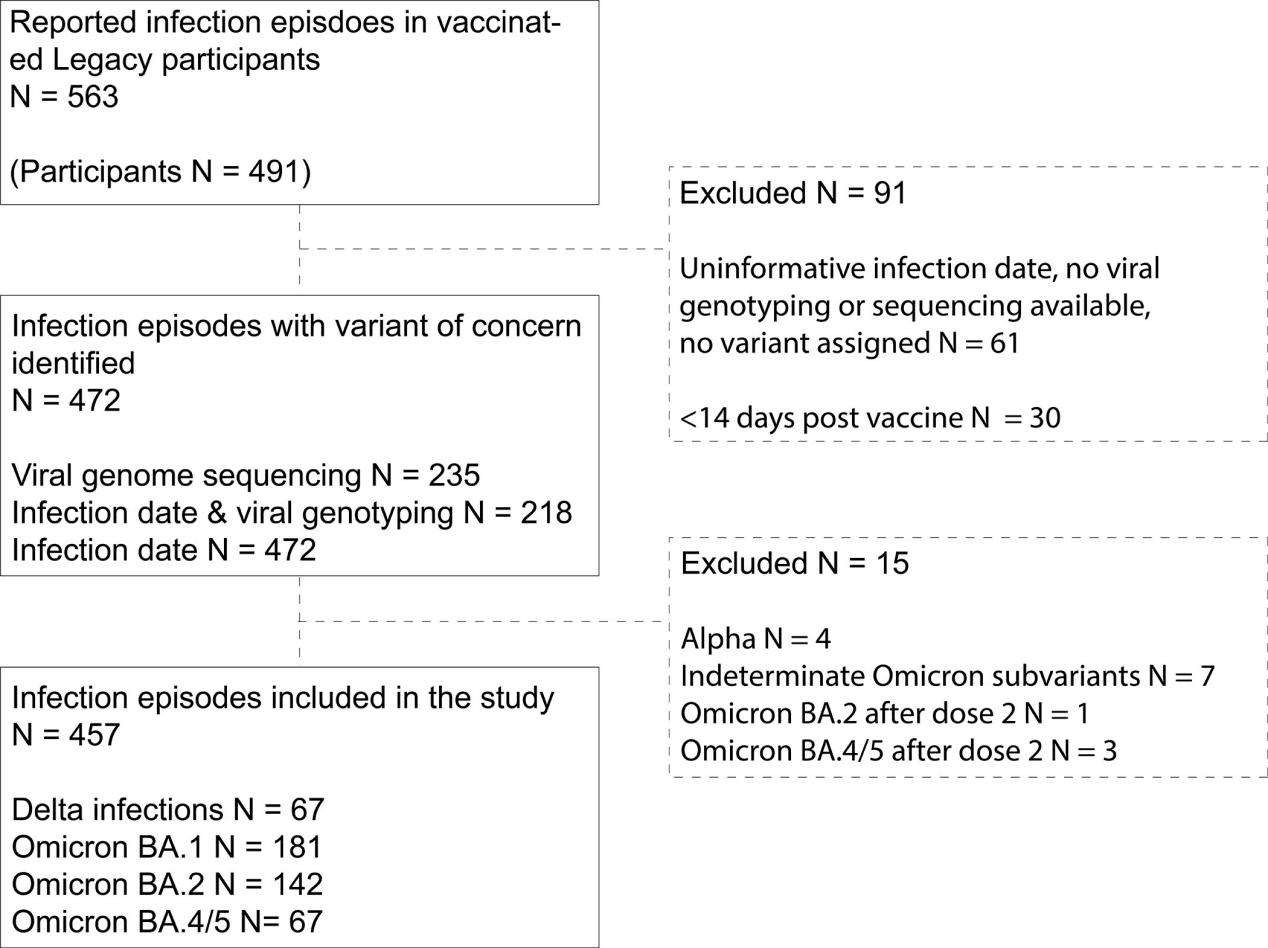
CONSORT diagram.

Inclusion and exclusion criteria for the wider *Legacy* study are as previously reported [5]. In summary, *Legacy* participants are adults age >18, employed by an institution that operated the UCLH-Crick asymptomatic PCR testing pipeline 2020–2022 [22], who gave written informed consent for active COVID-19 vaccination and infection surveillance. We included all adults in this study who reported a positive PCR test, either through the PCR surveillance or via home-based testing. All participants at the time of recruitment were undergoing mandatory weekly or twice weekly occupational health testing for COVID-19 [23] when required to be in-work. *Legacy* has minimal exclusion criteria, beyond those unable or unwilling to give informed consent, or not employed by an institution using the UCLH-Crick PCR testing pipeline. For this study we included study participants reporting an infection episode between 10^th^ June 2021 and 14^th^ September 2022 (Delta to Omicron BA.5 infection periods). We excluded infection episodes from analyses if their infection was ≤ 14 days after a vaccination, if < 14 days had passed between the infection date and date of data export (to mitigate against missing symptom diaries from recent infections). We reported observational data from all variant-specific cohorts but further excluded those cohorts with < 5 participants from the cross-variant analysis (**Fig 1**).

### Infection episodes

Infection episodes were defined as a positive SARS-CoV-2 test, either through asymptomatic occupational screening or following additional symptomatic testing using either PCR or antigen-based testing. Participants underwent mandatory occupational health screening between 1^st^ April 2020 and 31^st^ May 2022, we continued active voluntary surveillance beyond this to September 2022 to capture the period of BA.5 dominance, requesting participants report any symptomatic episodes, or asymptomatic lateral flow positive test results to the study team. Participants reporting an infection episode between June 2021 and September 2022 had same-day swabs collected by courier on alternate weekdays up to day 10 post symptom onset (defined as first day of any symptoms of any severity) or day 10 post positive swab, whichever was earlier. An additional swab where possible was performed between day 11 and day 30 after return to work. We excluded episodes from analysis if we were unable to determine the infecting variant: usually infections occurring at the transition of dominant VOCs without additional molecular testing (**Fig 1**). As the spike sequence of Omicron BA.4 and BA.5 are identical, we merged episodes assigned as BA.4 (by sequencing), BA.5 (by sequencing), or BA.4/5 (by PCR genotyping or date) into a single group: BA.4/5.

### Symptom reporting

Symptom severity in non-hospitalised adults was self-reported via an online symptom diary during the infective period. Participants self-reported symptoms using a standardised electronic symptom diary within REDCap. These data were verified by a study clinician at a study visit following the infection episode. To capture the scale of symptom severity experienced by participants, we assigned symptom severity categories to those with asymptomatic infection (0), mild (I), moderate (II) and severe (III), expanding the WHO categories 1–2 [24], in the absence of validated severity scores for non-hospitalised adults. Symptoms were defined as follows, grade I: “does not interfere with the participant’s daily routine and does not require further procedure; it causes slight discomfort”; grade II: “interferes with some aspects of the participant’s routine, or requires further procedure, but is not damaging to health; it causes moderate discomfort”; grade III: “results in alteration, discomfort or disability which is clearly damaging to health”. We further categorised symptom profiles in two ways, excluding individuals who had not completed a symptom diary. Firstly, into three categories based on the original NHS symptoms of COVID-19: symptomatic with one or more “classic” cardinal NHS symptoms (cough, fever, anosmia), symptomatic with only non-cardinal symptoms, or not symptomatic. Secondly, into four categories using the updated NHS & CDC guidance [1, 25] on the triggers for isolation with symptomatic SARS-CoV-2: asymptomatic, symptomatic and afebrile, febrile alone, and febrile with other symptoms.

### SARS-CoV-2 RT-qPCR and sequencing

RNA was extracted from self-performed upper respiratory tract dry swabs taken at time of breakthrough infection, as previously described [23]. Viral RNA was genotyped by RT-qPCR (TaqPath COVID-19 CE-IVD Kit, ThermoFisher) to confirm SARS-CoV-2 infection. This PCR assay was validated in house against known viral copy numbers, obtained from live-virus quantification [5, 23]. Viral RNA from positive swabs was prepared for whole-genome sequencing using the ARTIC method (https://www.protocols.io/view/ncov-2019-sequencing-protocol-v3-locost-bh42j8ye) and sequenced on the ONT GridION platform to >30k reads / sample. All swab processing for all study sites was performed in the same laboratory. The data were demultiplexed and processed using the viralrecon pipeline (https://github.com/nf-core/viralrecon). All sequencing data were uploaded to COG-UK and independently verified.

### Data curation

Study data were collected and managed using REDCap electronic data capture tools hosted at University College London. Identifying data were accessed by the clinical members of the study team (HT/JG/MD/ECW) for the purposes of communication with participants, study logistics including serial swab samples and post-infection study visits. Pseudonymised data were exported weekly from REDCap into R for rolling linkage with laboratory data, visualisation and analysis [26]. The remainder of the study team were only able to access this pseudonymised dataset for the purposes of data analysis. For this study, data were exported up to 28th September 2022 and the subsequent R record was locked.

### Data analysis, statistics and visualisation

Data were analysed in R (v 4.2.2). Summary descriptions of the clinical cohort and of reported symptoms and measured viral loads were generated, specifying calculation of median and IQR for continuous variables. Chi-squared testing was used for univariate comparisons of categorical variables, including individual symptoms by variant infection. For odds ratios and confidence intervals, we used the Wald normal approximation function *oddsratio*.*wald* from the *epitools* package. For the duration of symptoms and time-since-dose comparisons, infection episodes were grouped as above, and an unpaired two-tailed Wilcoxon test performed. Graphs were generated using the *ggplot2* package in R.

Hierarchical clustering was performed using the *pheatmap* package. Each infection episode’s symptom diary was reduced to presence / absence of each symptom, and data were subjected to unsupervised Euclidean clustering with Jaccard distances. The entire cohort was clustered together, and each variant clustered independently to explore per-variant differences in symptom patterns.

SARS-CoV-2 PCR data were analysed using the Cycle Threshold (Ct) of the ORF1ab gene target; smoothed spline fits were applied to Ct trajectories of all participants for each VOC. A correction of -1d was applied to original surveillance tests (but not serial swabs), assuming most surveillance tests were taken on the preceding evening. Peak Cts were drawn from the lowest Ct value (corresponding to the highest viral load) obtained from each participant between days 1–4. Ct values were compared between groups using an unpaired two tailed Wilcoxon test. Relationships between peak Ct values and time were estimated with linear regression coefficients (β) and reported with a 95% confidence interval (CI).

### Ethical approvals

The *Legacy* study was approved by London Camden and Kings Cross Health Research Authority Research and Ethics committee (Reference *20/HRA/4717* IRAS number 286469) and is sponsored by University College London Hospitals. All participants gave written informed consent on enrolment to the study, witnessed by a member of the clinical study team and recorded in REDCap. Participants were free to withdraw from the study at any time.

## Results

### Infection episodes

Infection following vaccination was reported in 563 episodes across 491 participants, resulting in a total of 1067 swabs analysed, with a median of 4 swabs per participant per infection episode. We were able to confidently determine the VOC that caused the infection in 472/563 (83%) of episodes using a combination of methods: by infection date relative to the dominant circulating VOC (472/563; 83%) [22], a combination of infection date and viral genotyping (218/563; 39%), or by viral genome sequencing (235/563; 42%) (**Fig 1**). We were unable to resolve the VOC in 61/563 episodes (11%) due to overlapping periods of VOC dominance and inconclusive or unavailable genomic data; these episodes were excluded from analysis. Cases within 14 days of vaccination did not meet the definition of post-vaccine infection and were excluded (30/563; 5%), as were four alpha infections (two determined by date; two by S gene target failure and date). The remaining 457 episodes, across 415 individuals, were then analysed (**Table 1**).

**Table 1:** Demographic details of legacy study participants with post-vaccine SARS-CoV-2 infection. The infecting variant and the number of preceding vaccinations are indicated at the top of each column.

Characteristic	Delta 2, N = 60^1^	Delta 3, N = 7^1^	Omicron-BA.1 2, N = 27^1^	Omicron-BA.1 3, N = 154^1^	Omicron-BA.2 3, N = 142^1^	Omicron-BA.4/5 3, N = 67^1^
**Dose 2**						
AZD1222	30 (50%)	0 (0%)	11 (41%)	35 (23%)	35 (25%)	20 (30%)
BNT162b2	29 (48%)	7 (100%)	13 (48%)	115 (75%)	99 (70%)	45 (67%)
mRNA1273	1 (1.7%)	0 (0%)	3 (11%)	4 (2.6%)	7 (4.9%)	2 (3.0%)
others	0 (0%)	0 (0%)	0 (0%)	0 (0%)	1 (0.7%)	0 (0%)
**Dose 3**						
AZD1222	0 (0%)	0 (0%)	0 (0%)	1 (0.6%)	0 (0%)	0 (0%)
BNT162b2	39 (76%)	7 (100%)	12 (92%)	142 (92%)	136 (96%)	58 (87%)
mRNA1273	12 (24%)	0 (0%)	1 (7.7%)	10 (6.5%)	6 (4.2%)	9 (13%)
Others	0 (0%)	0 (0%)	0 (0%)	1 (0.6%)	0 (0%)	0 (0%)
Unknown	9	0	14	0	0	0
**Site**						
NHS	14 (23%)	3 (42%)	3 (11%)	55 (35%)	35 (24%)	17 (25%)
Crick (non-NHS)	46 (77%)	4 (57%)	24 (89%)	99 (64%)	107 (75%)	50 (75%)
**Sex**						
Female	37 (62%)	4 (57%)	17 (63%)	107 (69%)	97 (68%)	51 (76%)
Male	23 (38%)	3 (43%)	10 (37%)	47 (31%)	45 (32%)	16 (24%)
**Age (years)**						
Median [IQR]	39 [28–48]	47 [40–52]	34 [29–40]	40 [32–49]	40 [31–48]	37 [29–51]
**Episode number**						
1	60 (100%)	6 (86%)	25 (93%)	149 (97%)	119 (84%)	42 (63%)
2	0 (0%)	1 (14%)	2 (7.4%)	5 (3.2%)	22 (15%)	25 (37%)
3	0 (0%)	0 (0%)	0 (0%)	0 (0%)	1 (0.7%)	0 (0%)
**Joined study before infection episode?**						
No	25 (42%)	5 (71%)	16 (59%)	55 (36%)	42 (30%)	13 (19%)
Yes	35 (58%)	2 (29%)	11 (41%)	99 (64%)	100 (70%)	54 (81%)
**Days since dose prior to infection**						
Median [IQR]	155 [110–192]	41 [27–57]	207 [166–263]	82 [52–106]	145 [103–177]	228 [193–277]
**Self-reported symptom severity**						
grade I	22 (38%)	0 (0%)	12 (48%)	55 (36%)	52 (40%)	21 (33%)
grade II	25 (43%)	0 (0%)	7 (28%)	56 (37%)	62 (48%)	29 (46%)
grade III	0 (0%)	0 (0%)	0 (0%)	2 (1.3%)	3 (2.3%)	1 (1.6%)
asymptomatic	11 (19%)	4 (100%)	6 (24%)	38 (25%)	12 (9.3%)	12 (19%)
Unknown	2	3	2	3	13	4
Self-reported duration of symptoms	9 [7–15]	3 [0–8]	10 [7–16]	9 [6–13]	8 [5–12]	7 [4–12]

^1^ n (%); Median [25%-75%]

These individuals had the same age distribution (median 39 years [IQR 31–49], as the whole *Legacy* study (median 40 years [31–50]), and were gender matched to *Legacy* (281 vs 535 female; 68 vs 68% female). Online symptom questionnaires were completed by participants for 430/457 episodes (94%) (**Table 1**).

### Variant-specific cohorts

**Fig 2:**
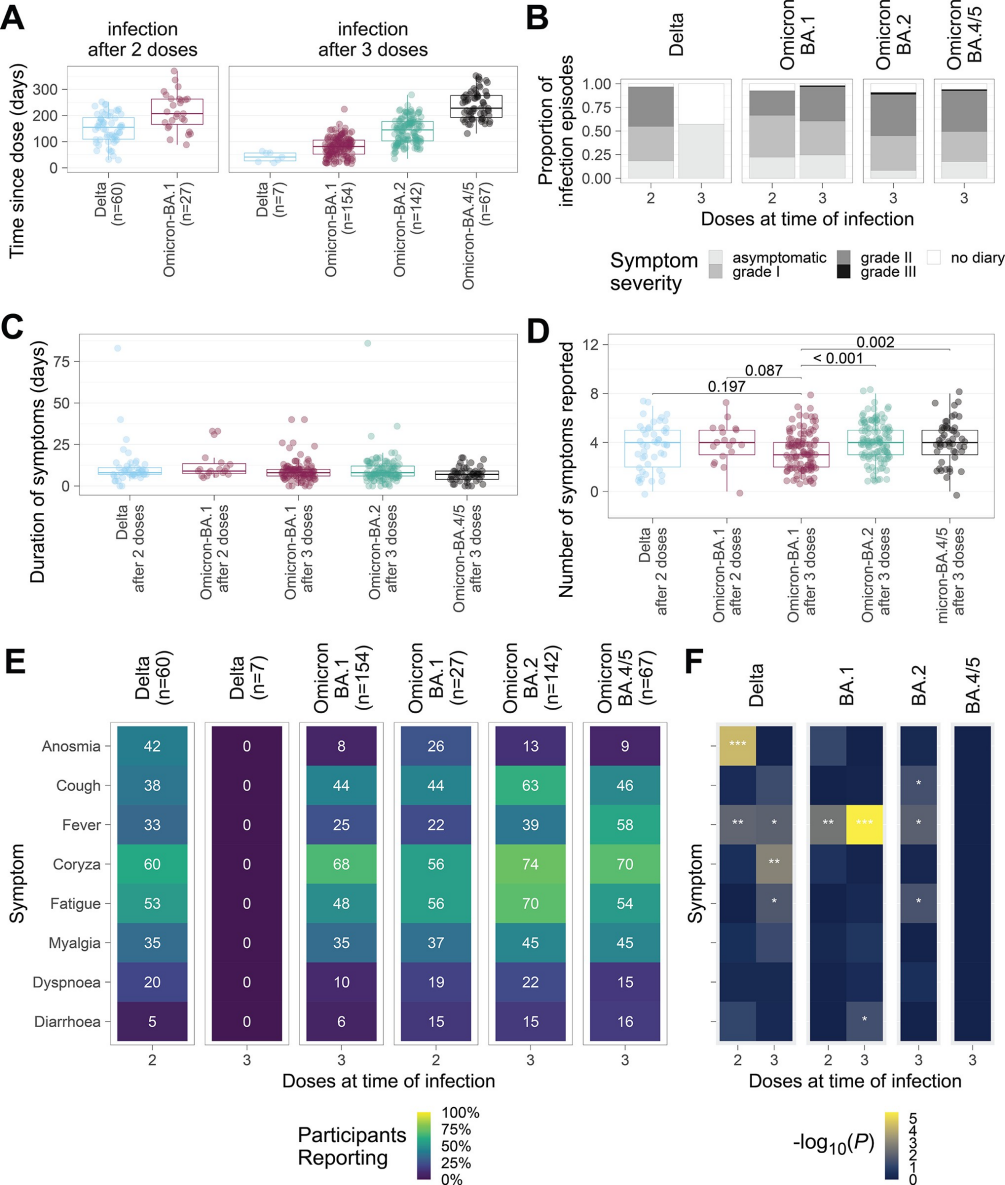
Symptoms of COVID-19 are an interaction between prevailing variants and vaccinations. (**A**) Time since vaccine dose (2 or 3) in days before the start of an infection episode for each variant of concern (VOC). (**B**) Proportion of participants reporting no, mild, moderate, or severe symptoms during their infection episode. (**C**) Duration of each infection episode in days stratified by the VOC and number of doses received prior to infection. (**D**) Number of symptoms reported by participants, stratified by VOC and number of doses received prior to infection (**E**) Percentage of individuals reporting each symptom is shown as a heatmap. Percentage shown in each tile, with the tiles shaded to reflect that percentage. The denominator used is all infection episodes of the corresponding VOC and number of doses. (**F**) Heatmap showing negative decimal logarithms of P values from χ2 tests comparing the presence/absence of a symptom between 3d-BA.4/5 (ref, reference) and the indicated infection episodes. Symptoms are ordered as in Fig 2E. Significant comparisons are marked as follows: P < 0.001 with ***; P < 0.01 with ** and P<0.05 with *.

We stratified infection episodes into six cohorts according to the dominant combinations of participant vaccination status and virus variant (**Fig 2A and Table 1**): Delta infection following 2 doses (2d+Delta: n = 60, occurring a median of 155 [IQR 109–194] days since last vaccine), Delta following 3 doses (3d+Delta: n = 7, 41 [IQR 26–56] days), Omicron BA.1 following 2 doses (2d+BA.1: n = 27, 207 [IQR 166–263] days), Omicron BA.1 following 3 doses (3d+BA.1: n = 154, 82 [IQR 52–106] days, Omicron BA.2 following 3 doses (3d+BA.2: n = 142, 145 [IQR 103–177]). Due the identical spike proteins of the Omicron subvariants BA.4 and BA.5, these infection episodes were considered a single group, Omicron BA.4/5 following 3 doses (3d+BA.4/5: N = 67, 228 [IQR 192–276] days).

### COVID-19 symptoms

While the majority of participants (347/457; 76%) reported grade I-II severity illness (**Fig 2B and S1 Table**), asymptomatic infections were observed in each cohort. Amongst participants with a symptom diary, we found the proportion of asymptomatic infection was significantly lower for BA.1 (44/176; 25%) compared to BA.2 episodes (12/129; 9%; χ^2^ test P < 0.001; OR 0.3 [95% CI 0.15–0.61]). BA.4/5 was similar to BA.1 with 19% of participants reporting asymptomatic infection (12/63; χ^2^ test P = 0.28; OR 1.5 [0.7–3.0]). Within the symptomatic participants, we compared both the duration and symptom number. In the symptomatic cohort, the median duration of symptoms was 8 [IQR 6–11] days. Symptom duration did not differ among any of the groups (**Fig 2C**). The median number of symptoms experienced, 4 [IQR 3–5], also showed little variation between groups (**Fig 2D**).

Although the duration and number of symptoms were not clearly variant-specific, there were significant changes in the reporting of anosmia between groups. Anosmia was reported in significantly fewer cases in the 3d+Omicron cohorts (8–13%) as compared to 2d+Delta (26/60; 42%; χ^2^ test P < 0.001; 2d+Delta vs. Omicron BA.1 or vs. BA.2, or BA.4/5, respectively) (**Fig 2E and S2 Table**). In addition, anosmia was less prevalent in Omicron BA.1 infections after 3 doses (12/154; 8%) compared with 2 doses (7/27; 26%; χ2 test P = 0.013; OR 0.2 [0.1–0.7]).

Additionally, cough was more frequently reported in 3d+Omicron BA.2 (89/142; 63%) compared to 2d+Delta (23/60; 38%; χ^2^ test P = 0.002; OR 2.7 [1.5–5]). Whereas fever was observed in the majority of 3d+BA.4/5 infections (39/67; 58%) compared to 33% for d3+Delta (20/60; χ2 test P = 0.008; OR 2.8 [1.4–5.7]) and <39% for the other Omicron subvariants (**Fig 2F**).

Despite reports of changes in tissue tropism in laboratory studies of Omicron BA.1 [27], the proportion of participants reporting coryza, fatigue, myalgia, shortness of breath, and diarrhoea remained broadly similar across all combinations of cohorts that reported symptomatic illness (2d+Delta, 2d+BA.1, 3d+BA.1, BA.2, BA.4/5).

### Symptom clusters

**Fig 3:**
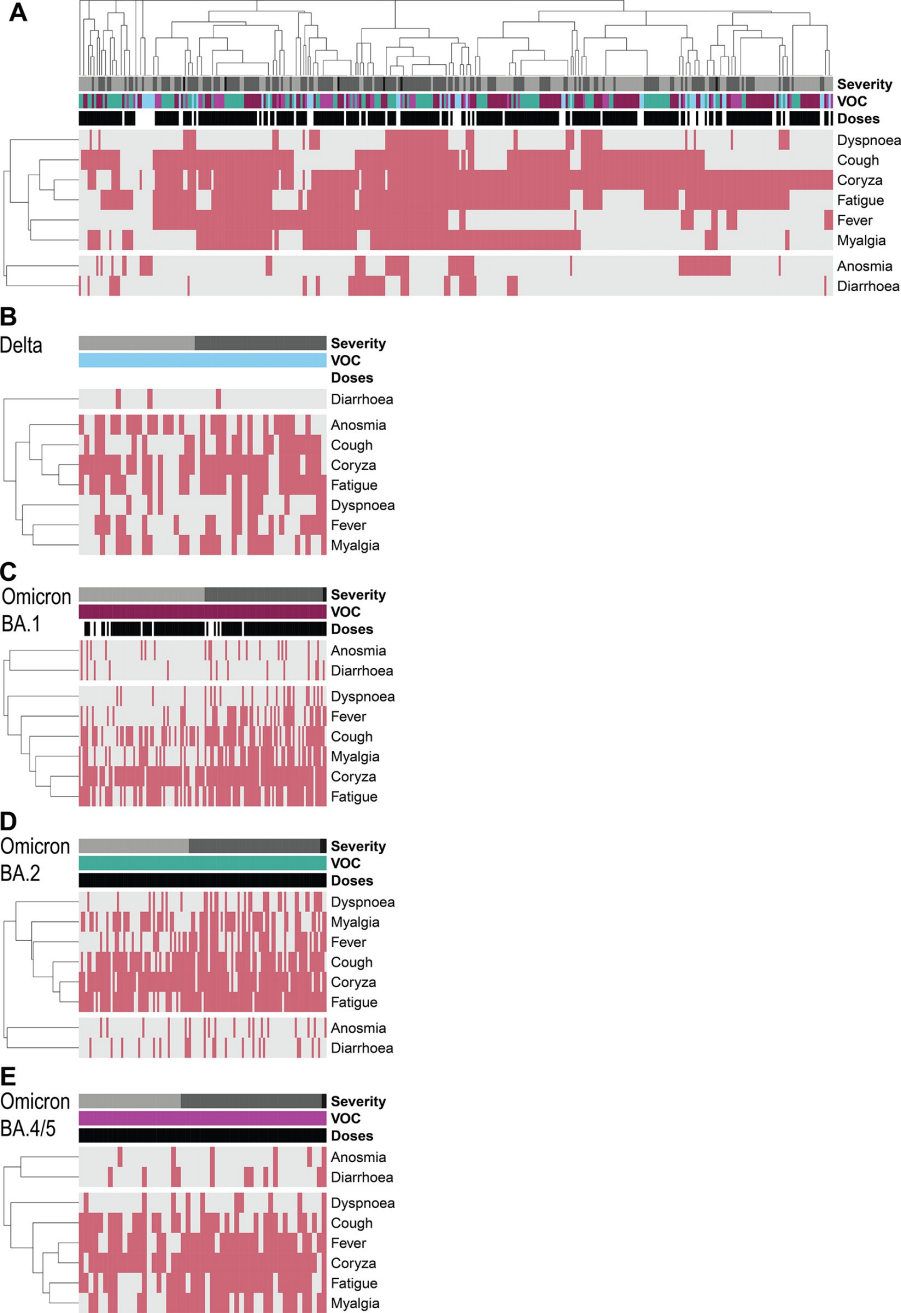
Hierarchical clustering of symptom patterns in infection episodes occurring after dose 2. All symptomatic episodes from the Legacy cohort clustered by individual (columns) and symptoms (rows) using Jaccard distances. The presence of a symptom is indicated by rose shading and the absence of a symptom by grey shading. Above each individual episode (each column) the colour bar indicates the severity, assigned VOC of that infection episode, and the number of doses of vaccine received before that infection. An individual may be present >1 if they experienced more than one infection episodes. Symptomatic episodes depicted across all VOCs in (A), all infection episodes, (B) Delta, (C) Omicron BA.1 (D) Omicron BA.2, (E) Omicron BA.4/5. Asymptomatic infection episodes are not shown.

We undertook a hierarchical clustering analysis of the symptom data to investigate which symptoms presented simultaneously in the differing contexts of variant and vaccine status (**Fig 3**). Symptom clusters were not apparent when all variants were considered in an unsupervised clustering of the whole study (**Fig 3A**). However, when symptoms were analysed for individual VOCs, we found some distinct patterns. While both coryza and fatigue clustered together in Delta infections, cough and fever were less likely to be reported together; myalgia was reported in a minority of cases (around one-third), and clustered with fever (**Fig 3B**). Symptoms caused by infections with Omicron BA.1 and BA.2 were dominated by clusters consisting of cough, coryza and fatigue; fever and myalgia were less common but did predominately occur in this same cluster (**Fig 3B and 3C**). In contrast, participants with BA.4/5 infections most frequently reported coryza, with fever, myalgia, and fatigue most commonly co-reported (**Fig 3D and 3E**). Of patients self-reporting fever, almost all experienced another symptom, with only 2 individuals reporting fever alone (one after 3d+BA.1, and one after 2d+Delta).

While most participants were not febrile (75% or 61% for 3d+BA.1 or 3d+BA.2), NHS guidance also recommends self-isolation if an individual feels too unwell to carry out their routine activities. On the assumption that those reporting moderate severity symptoms (grades II or III) would not be able to attend work under the April 2022 guidelines [25] and therefore would self-isolate, 44% of our cohort with active infection (3d+BA.1 [38%] or 3d+BA.2 [50%] 3d+BA.5 [45%]) would still not meet self-isolation criteria for either for fever or severity, and thus would enter social circulation whilst likely infectious.

### Infection dynamics

**Fig 4:**
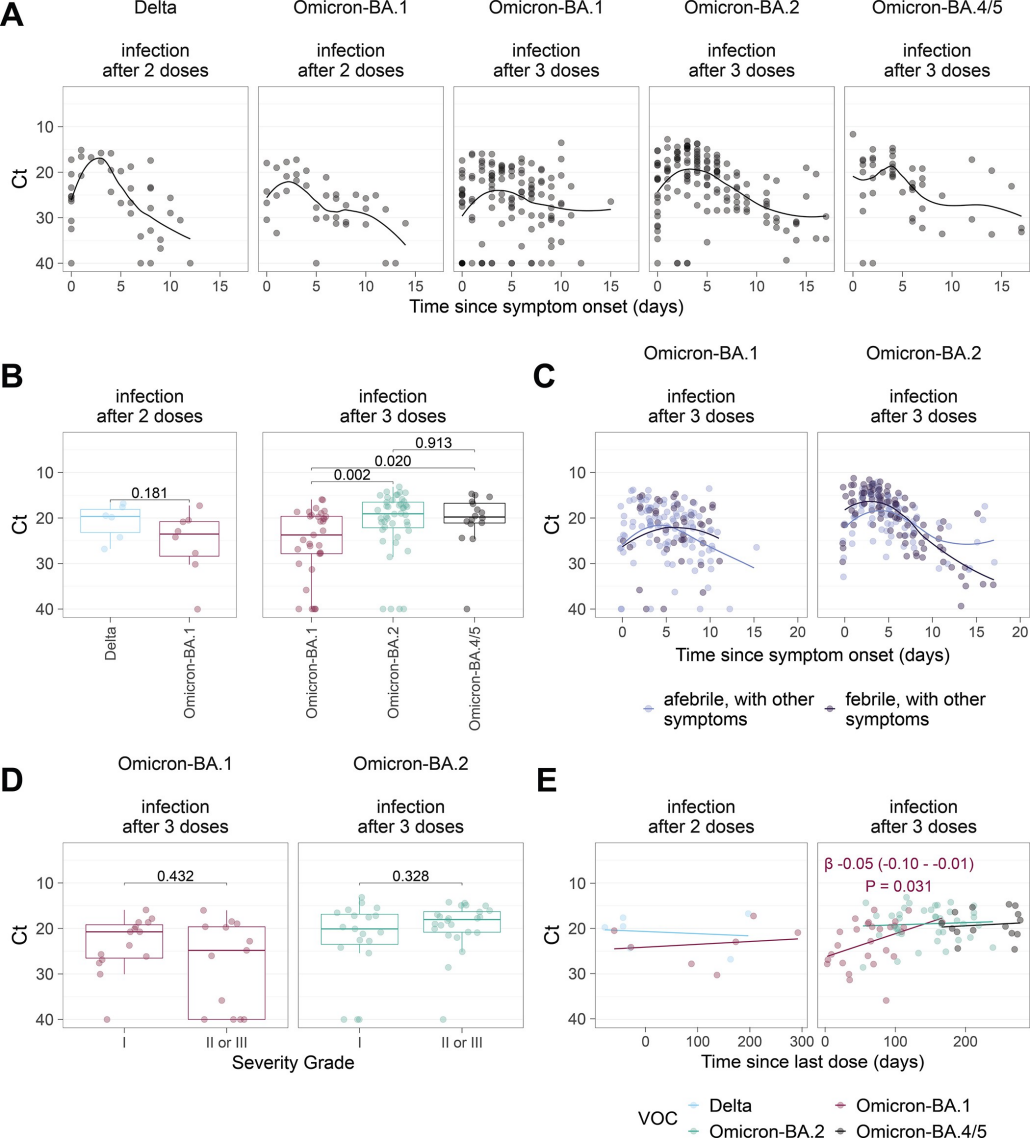
Peak viral load from symptomatic infection episodes in triple-vaccinated participants, compared to days since vaccination. (**A**) Viral load (Ct) trajectories (day 0 = symptom onset), plotted separately for each variant and stratified by the number of preceding vaccinations. Smoothed spline fits are shown. (**B**) Peak viral load on days 1–4 following symptom onset from Delta, Omicron BA.1, BA.2 and BA.4/5 infection episodes by vaccine dose number. (**C**) Viral load (Ct) trajectories for symptomatic BA.1 or BA.2 infections with febrile and afebrile infection episodes in dark or light blue respectively. Smoothed spline fits are shown. (**D**) Peak viral load on days 1–4 in participants with either BA.1 or BA.2 reported by symptom severity grade. (**E**) Peak Ct value across Delta, BA.1, BA.2 and BA.4/5 plotted against the time in days since last vaccine dose after either two or three vaccine doses. Lines from linear regression are shown with a coefficient (β), 95% confidence interval and P value for significant (P < 0.05) estimates.

To test if symptoms were associated with viral replication, we examined infection dynamics in more detail. Participants who reported acute infection provided serial self-performed upper respiratory tract swabs for RT-qPCR analysis of SARS-CoV-2 RNA during isolation, with predominately Delta, BA.1 and BA.2 infections. We profiled the kinetics of each infection using the Ct value, as an inverse proxy for representative of levels of replicating, viable virus [28, 29]. Across all cohorts, the median Ct values remained at levels considered to be infectious for 7–10 days, irrespective of symptom severity and including asymptomatic participants (**Fig 4A**). The lowest Ct values (corresponding to estimated peak viral load [28], hereafter referred to as peak) were observed between 2–5 days after symptom onset, with similar Ct trajectories observed across all VOCs tested. After 3 doses, peak Ct values were significantly higher in Omicron BA.1 infections (median minimum Ct 23.7) compared with BA.2 (median minimum Ct 19.1; Wilcoxon test P = 0.002) and BA.4/5 (median minimum Ct 19.8; Wilcoxon test P = 0.02) **(Fig 4B**). We then examined if dynamic and peak Ct values differed between those with fever and those who were symptomatic though afebrile. We found virtually identical viral load trajectories in those participants where we had adequate serial sampling Omicron BA.1 and BA.2 (**Fig 4C**).

Notably, there was also no significant difference in peak Ct values in those who met the NHS criteria for isolation and those who did not. Amongst symptomatic individuals, the presence of fever did not significantly affect the peak value for either BA.1 or BA.2 infections: median minimum Ct febrile vs. afebrile BA.1 (26.9 vs 20.7; Wilcoxon test P = 0.28) and BA.2 (16.9 vs 17.8; Wilcoxon test P = 0.16) (**S1 Fig**). Furthermore, the self-reported severity grade was not associated with differences in peak Ct for either BA.1 (median minimum Ct grade I vs grades II-III; 20.7 vs 24.8; Wilcoxon test P = 0.43) or BA.2 (median minimum Ct grade I vs grades II-III; 20.1 vs 18.0; Wilcoxon test P = 0.33) (**Fig 4D**). We did detect a trend towards lower Ct and increasing time since last vaccination dose that was significant for BA.1 infection after 3 doses (β = -0.05 (CI -0.10–0.01); P = 0.031) (**Fig 4E**), but this did not reach statistical significance for the other variants (all P > 0.05) (**S3 Table**).

## Discussion

Our large, longitudinal cohort study demonstrates the evolution of symptom profiles between Delta and Omicron sub-variants, including Omicron BA.4/5. In contrast, we also show the relatively unchanging peak viral loads in the respiratory tract, regardless of variant or vaccine history. Despite analyses of hospitalisation and mortality data indicating that Omicron caused less-severe clinical disease [8, 9, 14], our data suggests that COVID-19 caused by all Omicron sub-variants caused a significant symptom burden in community infections, with attendant impacts on healthcare resources and the economic impact of increased time off due to illness. In the absence of vaccines that generate sterilising immunity, continued infections with new variants, are likely to contribute to the increasing prevalence of post-COVID syndrome (PCS) or long COVID [16–18, 30].

Due to the nature of occupational health PCR screening in the *Legacy* cohort, we were able to include true asymptomatic infections within our analysis (25% of episodes of BA.1 after 3 doses), contrasting with cohort studies relying on symptom-triggered testing [19, 31, 32]. We found all cases of Delta infection following three vaccine doses were asymptomatic, contrasting with participants infected with Omicron sub-variants, who were more likely to be symptomatic despite a similar 3-month interval since vaccination and near-identical viral load trajectories across VOCs. The Delta comparator group was relatively small, but our data suggest that the immediate boosting effect of third vaccine dose may minimise symptoms, but this protection is short-lived. We found more individuals were vulnerable to symptomatic disease at the longer post-dose intervals when BA.2 and then BA.4/5 emerged which may be related to waning of vaccine-induced immunity [19, 33, 34]. Furthermore, we show an association between increasing time since vaccination and increasing viral loads in BA.1 infections, not captured by previous studies, that suggests waning mucosal, as well as humoral immunity may be exploited by SARS-CoV-2 [19, 20, 35].

Understanding changing symptomatology of SARS-CoV-2 variants is essential to inform public health messaging and testing guidance. We show anosmia, a key and relatively specific symptom of earlier SARS-COV-2 variants, is less common across the Omicron sub-variants to BA.4/5 than in Delta. This replicates the findings of large community based studies in the UK (ZOE, REACT) and the USA [19, 21, 31, 36], where reduction in anosmia incidence is the most notable difference between Delta and Omicron infections to BA.2. Accumulating vaccine doses may temporarily increase mucosal IgA and neutralising antibodies, further attenuating the damaging effects of viral replication in the olfactory cells [35, 37]. However, other upper respiratory symptoms and associated viral kinetics remained unchanged across variants irrespective of vaccine status, and thus it is unlikely that vaccine-induced mucosal antibodies significantly affect viral replication across the wider nasal epithelial, or deeper respiratory, surfaces.

Fever has been shown in other studies to be less frequently reported in infection episodes with Omicron BA.1/2 [19, 31]. We replicated this finding in our study, however, we found fever frequency was subsequently increased in Omicron BA.4/5 infections when compared to Delta infections. Other studies reporting variant-specific symptom profiles have not extended reporting to BA.4/5 infections, including the REACT study [31]; and the app-based ZOE study [19]. Our finding of increased fever with BA.4/5 infections is in line with prospective population level data recently reported from Japan [32]. Why fever may be more common with BA.4/5 infections is unknown, but waning immunity and increasing antigenic divergence may both impact on increasing symptom severity.

It is not fully clear from our data to what extent vaccination may suppress viral replication and transmission. We observed almost identical viral load trajectories across VOCs, irrespective of vaccination status and time since vaccination which corresponded closely with those found during both controlled human challenge models in unvaccinated individuals, asymptomatic household transmission in South Africa and healthcare workers in Turkey [20, 28, 29], suggesting that immunity induced by first-generation vaccines encoding an ancestral Spike might have minimal impact on VOC replication in the nasopharynx. However, we also observed a trend towards higher peak viral loads after longer time since vaccination that reached significance in BA.1 infections, mirroring waning neutralising antibodies in vaccinated cohorts [38]. These results are consistent with reduced transmission inferred from household attack rates in vaccinated compared to unvaccinated cohorts [39].

Due to the rapid evolution of SARS-CoV-2 across 2022, in combination with changing national vaccination policy, our data are subject to important limitations. Firstly, while we were able to obtain detailed prospective clinical and PCR data from our cohort across four waves of infection, we were not able to compare these data with earlier waves (i.e. Alpha/B.1.1.7, EU1/B.1.177, and D614G/B.1) as these preceded our study period of enhanced infection surveillance and symptom recall will differ from contemporaneous symptom diaries. Secondly, we were unable to control for differences in clinical baseline demographics between infection groups. However, *Legacy* participants are representative of healthy working age adults in London, as less than 10% of our cohort have significant clinical co-morbidities [5]. Thirdly, the symptom data are intrinsically subjective and will be influenced by a participant’s attitudes to and experiences and perceptions of health and illness. For example, an individual’s interpretation of “cold like symptoms” is likely to differ. Similar to population-based studies, we found a non-significant trend towards increasing coryza symptoms in both BA.2 and BA.4/5 infections. These larger studies were able to analyse specific upper respiratory symptoms, where participants reported higher rates of symptoms in keeping with coryza in Omicron BA.1 and BA.2, with ‘sneezing’, ‘sore throat’ and ‘runny nose’ reaching significance individually [31] and the ZOE study where ‘sneezing’ and ‘runny nose’ were associated with Delta infections, whereas ‘sore throat’ and ‘hoarse voice’ were more likely to occur in Omicron BA.1 infections [19, 31]. While symptom profiles and severity were self-reported during infection, all diaries were checked for accuracy with the participant by a study clinician within 21 days of reported symptom onset to minimise recall bias.

A fourth potential limitation is that our serial PCR testing was not supervised by clinician. Participants were given clear instructions on testing and had been compliant with asymptomatic screening for excess of 12 months prior to the start of this study, so sampling variability is likely to have a minimal effect on our results [40]. The viral load trajectories we observed were remarkably consistent and closely mirrored supervised testing in the SARS-CoV-2 human challenge study [28], suggesting high quality samples were obtained at serial time points across participants. The PCR assay used was validated in house against known viral copy numbers, obtained from live-virus quantification [5, 23]. Our reported Ct values are consistent with Omicron in the general population [41], and are well within the range in which infectious virus could be detected during both human challenge with SARS-CoV-2 and other prospectively sampled cohortsr [28, 29, 42]. The heterogenity in lowest value and longitudinal Ct kinetics in our cohort are also very similar to others [43, 44]. Finally, it is possible that the infecting variant has been mis-assigned, or there is confounding from excluding episodes that were not assigned a variant. This is also a challenge for the large cohort studies such as ZOE, reliant on test positivity reporting by participants, where the infecting variant is inferred from date of infection alone. In our study, we obtained sequencing confirmation and/or viral genotyping (by S gene target failure) in 59% of episodes, relying on episode date for only 41% of assignments. A strength of this analysis, compared to ZOE and similar is that nearly two-thirds of our variant assignments are supported directly by molecular testing.

In conclusion, we show that symptoms experienced by vaccinated adults are likely to change with new SARS-CoV-2 VOCs including within defined lineages such as Omicron BA.1-5. Guidance on self-isolation and testing requires regular evaluation from prospective clinical studies as new VOCs emerge, and notably, neither symptom severity nor presence of fever is a useful proxy for testing when considering the need for self-isolation. Updated advice should continue to emphasise the ongoing risk of transmission from individuals with no or mild symptoms whilst infected with Omicron to vulnerable populations, and the possibility for characteristic symptoms to change in the future were a new VOC to emerge.

## Supporting information

S1 Fig Peak viral load on days 1–4 following symptom onset from Omicron BA.1 and BA.2 by febrile and afebrile infection episodes in dark or light blue respectively. (DOCX)

S1 Table Composition of infection episodes by severity by count (N) and as a percentage (%) excluding episodes without a symptom diary (no diary). (DOCX)

S2 Table Chi-squared tests comparing the presence/absence of a symptom between d3-Omicron BA.4/5 (ref., reference) and the indicated variant of concern (VOC), with symptom count (N), number of episodes (Total), proportion (Prop.), odds ratio (OR) with 95% confidence interval (lower, upper). (DOCX)

S3 Table Regression of time since last vaccine dose (in days) on peak Ct value by variant of concern (VOC). Univariate linear regression coefficients (estimate) are shown with standard error (std.error) and associated P values.
(DOCX)
