# Supplementary material for: COVID-19 in non-hospitalised adults caused by either SARS-CoV-2 sub-variants Omicron BA.1, BA.2, BA.4/5 or Delta associates with similar illness duration, symptom severity and viral kinetics, irrespective of vaccination history
Source: PLoS One. 2024 Mar 21;19(3):e0294897. doi: 10.1371/journal.pone.0294897 (PMC10956747; doi:10.1371/journal.pone.0294897)
Supplement: S1 Fig — (DOCX) [file pone.0294897.s001.docx]

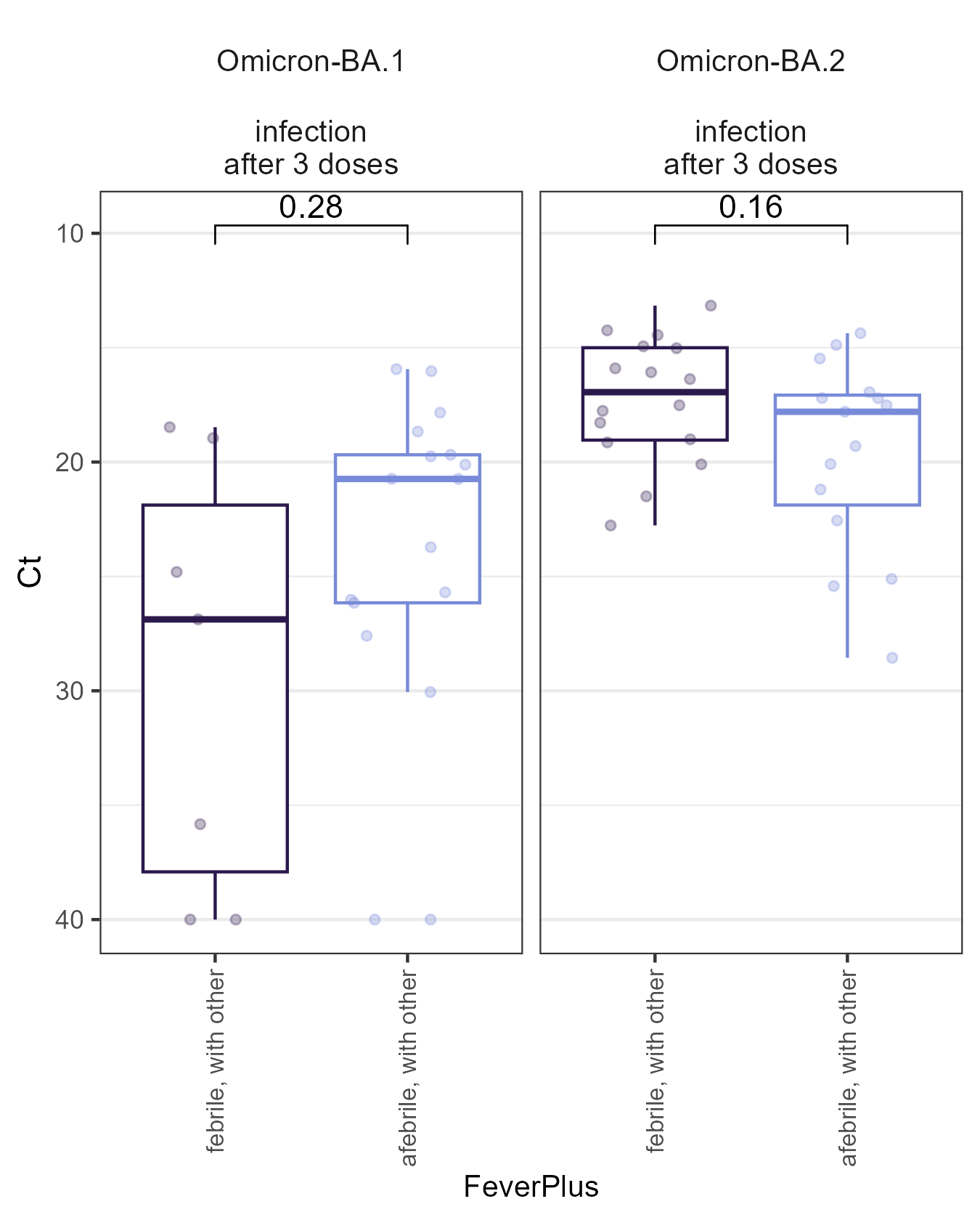


**Supplementary Figure 1** Peak viral load on days 1-4 following symptom onset from Omicron BA.1 and BA.2 by febrile and afebrile infection episodes in dark or light blue respectively.
