# Supplementary material for: COVID-19 in non-hospitalised adults caused by either SARS-CoV-2 sub-variants Omicron BA.1, BA.2, BA.4/5 or Delta associates with similar illness duration, symptom severity and viral kinetics, irrespective of vaccination history
Source: PLoS One. 2024 Mar 21;19(3):e0294897. doi: 10.1371/journal.pone.0294897 (PMC10956747; doi:10.1371/journal.pone.0294897)
Supplement: S1 Table — (DOCX) [file pone.0294897.s002.docx]

| Supplementary table 1: composition of infection events by severity | | | | | | | | | |
| --- | --- | --- | --- | --- | --- | --- | --- | --- | --- |
| VOC | asymptomatic (N) | grade I (N) | grade II (N) | grade III  (N) | asymptomatic (%) | grade I  (%) | grade II  (%) | grade III  (%) | no diary (N) |
| Delta | 15 | 22 | 25 | 0 | 24.19 | 35.48 | 40.32 | 0 | 5 |
| Omicron-BA.1 | 44 | 67 | 63 | 2 | 25 | 38.07 | 35.8 | 1.14 | 5 |
| Omicron-BA.2 | 12 | 52 | 62 | 3 | 9.3 | 40.31 | 48.06 | 2.33 | 14 |
| Omicron-BA.4/5 | 12 | 21 | 29 | 1 | 19.05 | 33.33 | 46.03 | 1.59 | 4 |

Composition of infection episodes by severity by count (N) and as a percentage (%) excluding episodes without a symptom diary (no diary).
