# Supplementary material for: COVID-19 in non-hospitalised adults caused by either SARS-CoV-2 sub-variants Omicron BA.1, BA.2, BA.4/5 or Delta associates with similar illness duration, symptom severity and viral kinetics, irrespective of vaccination history
Source: PLoS One. 2024 Mar 21;19(3):e0294897. doi: 10.1371/journal.pone.0294897 (PMC10956747; doi:10.1371/journal.pone.0294897)
Supplement: S2 Table — (DOCX) [file pone.0294897.s003.docx]

| supplementary table 2: Symptom profile by infecting variant and vaccine history | | | | | | | | | | | | |
| --- | --- | --- | --- | --- | --- | --- | --- | --- | --- | --- | --- | --- |
| VOC | Ref. | Symptom | N | Total | Prop. | Ref. N | Ref. Total | Ref. Prop | P value | OR | Lower | Upper |
| d2+Delta | d3+Omicron-BA.4/5 | Coryza | 36 | 60 | 0.6 | 47 | 67 | 0.7 | 0.311 | 0.64 | 0.31 | 1.33 |
| d2+Delta | d3+Omicron-BA.4/5 | Fatigue | 32 | 60 | 0.53 | 36 | 67 | 0.54 | 1 | 0.98 | 0.49 | 1.98 |
| d2+Delta | d3+Omicron-BA.4/5 | Myalgia | 21 | 60 | 0.35 | 30 | 67 | 0.45 | 0.347 | 0.66 | 0.32 | 1.36 |
| d2+Delta | d3+Omicron-BA.4/5 | Anosmia | 25 | 60 | 0.42 | 6 | 67 | 0.09 | < 0.001 | 7.26 | 2.72 | 19.41 |
| d2+Delta | d3+Omicron-BA.4/5 | Cough | 23 | 60 | 0.38 | 31 | 67 | 0.46 | 0.469 | 0.72 | 0.36 | 1.47 |
| d2+Delta | d3+Omicron-BA.4/5 | Fever | 20 | 60 | 0.33 | 39 | 67 | 0.58 | 0.009 | 0.36 | 0.17 | 0.74 |
| d2+Delta | d3+Omicron-BA.4/5 | Dyspnoea | 12 | 60 | 0.2 | 10 | 67 | 0.15 | 0.603 | 1.43 | 0.57 | 3.59 |
| d2+Delta | d3+Omicron-BA.4/5 | Diarrhoea | 3 | 60 | 0.05 | 11 | 67 | 0.16 | 0.077 | 0.27 | 0.07 | 1.01 |
| d2+Omicron-BA.1 | d3+Omicron-BA.4/5 | Coryza | 15 | 27 | 0.56 | 47 | 67 | 0.7 | 0.267 | 0.53 | 0.21 | 1.34 |
| d2+Omicron-BA.1 | d3+Omicron-BA.4/5 | Fatigue | 15 | 27 | 0.56 | 36 | 67 | 0.54 | 1 | 1.08 | 0.44 | 2.64 |
| d2+Omicron-BA.1 | d3+Omicron-BA.4/5 | Myalgia | 10 | 27 | 0.37 | 30 | 67 | 0.45 | 0.648 | 0.73 | 0.29 | 1.82 |
| d2+Omicron-BA.1 | d3+Omicron-BA.4/5 | Anosmia | 7 | 27 | 0.26 | 6 | 67 | 0.09 | 0.068 | 3.56 | 1.07 | 11.83 |
| d2+Omicron-BA.1 | d3+Omicron-BA.4/5 | Cough | 12 | 27 | 0.44 | 31 | 67 | 0.46 | 1 | 0.93 | 0.38 | 2.28 |
| d2+Omicron-BA.1 | d3+Omicron-BA.4/5 | Fever | 6 | 27 | 0.22 | 39 | 67 | 0.58 | 0.003 | 0.21 | 0.07 | 0.57 |
| d2+Omicron-BA.1 | d3+Omicron-BA.4/5 | Dyspnoea | 5 | 27 | 0.19 | 10 | 67 | 0.15 | 0.905 | 1.3 | 0.4 | 4.22 |
| d2+Omicron-BA.1 | d3+Omicron-BA.4/5 | Diarrhoea | 4 | 27 | 0.15 | 11 | 67 | 0.16 | 1 | 0.89 | 0.26 | 3.07 |
| d3+Delta | d3+Omicron-BA.4/5 | Coryza | 0 | 7 | 0 | 47 | 67 | 0.7 | 0.001 | 0 | 0 | NaN |
| d3+Delta | d3+Omicron-BA.4/5 | Fatigue | 0 | 7 | 0 | 36 | 67 | 0.54 | 0.021 | 0 | 0 | NaN |
| d3+Delta | d3+Omicron-BA.4/5 | Myalgia | 0 | 7 | 0 | 30 | 67 | 0.45 | 0.059 | 0 | 0 | NaN |
| d3+Delta | d3+Omicron-BA.4/5 | Anosmia | 0 | 7 | 0 | 6 | 67 | 0.09 | 0.922 | 0 | 0 | NaN |
| d3+Delta | d3+Omicron-BA.4/5 | Cough | 0 | 7 | 0 | 31 | 67 | 0.46 | 0.05 | 0 | 0 | NaN |
| d3+Delta | d3+Omicron-BA.4/5 | Fever | 0 | 7 | 0 | 39 | 67 | 0.58 | 0.011 | 0 | 0 | NaN |
| d3+Delta | d3+Omicron-BA.4/5 | Dyspnoea | 0 | 7 | 0 | 10 | 67 | 0.15 | 0.604 | 0 | 0 | NaN |
| d3+Delta | d3+Omicron-BA.4/5 | Diarrhoea | 0 | 7 | 0 | 11 | 67 | 0.16 | 0.546 | 0 | 0 | NaN |
| d3+Omicron-BA.1 | d3+Omicron-BA.4/5 | Coryza | 104 | 154 | 0.68 | 47 | 67 | 0.7 | 0.82 | 0.89 | 0.47 | 1.65 |
| d3+Omicron-BA.1 | d3+Omicron-BA.4/5 | Fatigue | 74 | 154 | 0.48 | 36 | 67 | 0.54 | 0.529 | 0.8 | 0.45 | 1.42 |
| d3+Omicron-BA.1 | d3+Omicron-BA.4/5 | Myalgia | 54 | 154 | 0.35 | 30 | 67 | 0.45 | 0.224 | 0.67 | 0.37 | 1.19 |
| d3+Omicron-BA.1 | d3+Omicron-BA.4/5 | Anosmia | 12 | 154 | 0.08 | 6 | 67 | 0.09 | 0.982 | 0.86 | 0.31 | 2.39 |
| d3+Omicron-BA.1 | d3+Omicron-BA.4/5 | Cough | 67 | 154 | 0.44 | 31 | 67 | 0.46 | 0.816 | 0.89 | 0.5 | 1.59 |
| d3+Omicron-BA.1 | d3+Omicron-BA.4/5 | Fever | 38 | 154 | 0.25 | 39 | 67 | 0.58 | < 0.001 | 0.24 | 0.13 | 0.43 |
| d3+Omicron-BA.1 | d3+Omicron-BA.4/5 | Dyspnoea | 16 | 154 | 0.1 | 10 | 67 | 0.15 | 0.462 | 0.66 | 0.28 | 1.54 |
| d3+Omicron-BA.1 | d3+Omicron-BA.4/5 | Diarrhoea | 10 | 154 | 0.06 | 11 | 67 | 0.16 | 0.039 | 0.35 | 0.14 | 0.88 |
| d3+Omicron-BA.2 | d3+Omicron-BA.4/5 | Coryza | 105 | 142 | 0.74 | 47 | 67 | 0.7 | 0.683 | 1.21 | 0.63 | 2.3 |
| d3+Omicron-BA.2 | d3+Omicron-BA.4/5 | Fatigue | 100 | 142 | 0.7 | 36 | 67 | 0.54 | 0.027 | 2.05 | 1.12 | 3.74 |
| d3+Omicron-BA.2 | d3+Omicron-BA.4/5 | Myalgia | 64 | 142 | 0.45 | 30 | 67 | 0.45 | 1 | 1.01 | 0.56 | 1.81 |
| d3+Omicron-BA.2 | d3+Omicron-BA.4/5 | Anosmia | 18 | 142 | 0.13 | 6 | 67 | 0.09 | 0.579 | 1.48 | 0.56 | 3.91 |
| d3+Omicron-BA.2 | d3+Omicron-BA.4/5 | Cough | 89 | 142 | 0.63 | 31 | 67 | 0.46 | 0.037 | 1.95 | 1.08 | 3.51 |
| d3+Omicron-BA.2 | d3+Omicron-BA.4/5 | Fever | 55 | 142 | 0.39 | 39 | 67 | 0.58 | 0.013 | 0.45 | 0.25 | 0.82 |
| d3+Omicron-BA.2 | d3+Omicron-BA.4/5 | Dyspnoea | 31 | 142 | 0.22 | 10 | 67 | 0.15 | 0.324 | 1.59 | 0.73 | 3.48 |
| d3+Omicron-BA.2 | d3+Omicron-BA.4/5 | Diarrhoea | 22 | 142 | 0.15 | 11 | 67 | 0.16 | 1 | 0.93 | 0.42 | 2.06 |

Chi-squared tests comparing the presence/absence of a symptom between d3-Omicron BA.4/5 (ref., reference) and the indicated variant of concern (VOC), with symptom count (N), number of episodes (Total), proportion (Prop.), odds ratio (OR) with 95% confidence interval (lower, upper).
