# Supplementary material for: COVID-19 in non-hospitalised adults caused by either SARS-CoV-2 sub-variants Omicron BA.1, BA.2, BA.4/5 or Delta associates with similar illness duration, symptom severity and viral kinetics, irrespective of vaccination history
Source: PLoS One. 2024 Mar 21;19(3):e0294897. doi: 10.1371/journal.pone.0294897 (PMC10956747; doi:10.1371/journal.pone.0294897)
Supplement: S3 Table — Univariate linear regression coefficients (estimate) are shown with standard error (std.error) and associated P values. (DOCX) [file pone.0294897.s004.docx]

| supplementary table 3: relationship between peak Ct values, vaccination history and infecting variant of concern | | | | | | | | |
| --- | --- | --- | --- | --- | --- | --- | --- | --- |
| VOC | term | estimate | std.error | statistic | p.value | lower | upper | CI |
| 2d+Delta | daysSincedose | 0 | 0.016262 | 0.292465 | 0.784 | -0.03 | 0.04 | (-0.03 - 0.04) |
| 2d+Omicron-BA.1 | daysSincedose | -0.01 | 0.015549 | -0.39925 | 0.706 | -0.04 | 0.02 | (-0.04 - 0.02) |
| 3d+Omicron-BA.1 | daysSincedose | -0.05 | 0.02255 | -2.27626 | 0.031 | -0.1 | -0.01 | (-0.10 - -0.01) |
| 3d+Omicron-BA.2 | daysSincedose | 0 | 0.012656 | -0.3894 | 0.699 | -0.03 | 0.02 | (-0.03 - 0.02) |
| 3d+Omicron-BA.4/5 | daysSincedose | -0.01 | 0.020195 | -0.3948 | 0.699 | -0.05 | 0.03 | (-0.05 - 0.03) |

Regression of time since last vaccine dose (in days) on Peak Ct value by variant of concern (VOC). Univariate linear regression coefficients (estimate) are shown with standard error (std.error) and associated P values.
